# Expression profiles of itch markers during scabies infection in a porcine scabies model

**DOI:** 10.1371/journal.pntd.0013788

**Published:** 2025-12-01

**Authors:** Deepani D. Fernando, Jakub O'Grady, Tam H. Nguyen, Gunter Hartel, Gil Yosipovitch, Katja Fischer

**Affiliations:** 1 Infection and Inflammation Program, QIMR Berghofer, Brisbane, Queensland, Australia; 2 School of Veterinary Science, University of Queensland, Gatton, Queensland, Australia; 3 Microscopy and Spatial Cell Biology Facility, QIMR Berghofer, Brisbane, Queensland, Australia; 4 Population Health Program, QIMR Berghofer, Brisbane, Queensland, Australia; 5 University of Miami Miller School of Medicine, Dr Phillip Frost Department of Dermatology and Cutaneous Surgery and Miami Itch Center, Miami, Florida, United States of America; London School of Hygiene and Tropical Medicine, UNITED KINGDOM OF GREAT BRITAIN AND NORTHERN IRELAND

## Abstract

**Background:**

Scabies is a debilitating parasitic skin disease caused by the obligate parasitic mite *Sarcoptes scabiei*, which inhabits the upper layers of the epidermis. In over 90% of affected individuals intense itching is the primary clinical symptom. This is accompanied by other delayed-type hypersensitivity responses. Persistent scratching due to the itch compromises skin integrity, facilitating the entry of pathogenic bacteria. Combined with immune-modulatory factors secreted by the mites, this creates a permissive environment for bacterial colonisation often leading to secondary complications. Effective treatment of scabies-associated itch could significantly reduce these complications and improve patients’ quality of life. However, current antipruritic therapies are inadequate: in most cases, antihistamines provide little relief for scabies-related itch, and systemic corticosteroids are generally contraindicated. Thus, there is a critical need for targeted therapies, yet progress has been hindered by a limited understanding of the underlying pathobiology of scabies-induced pruritus.

**Methodology/Principal Findings:**

We investigated the expression of itch-associated mediators - encompassing both histaminergic and non-histaminergic pathways - using immunohistochemistry in a porcine scabies model. Skin biopsies were collected at multiple time points during the course of infection. We observed upregulated expression of key non-histaminergic itch receptors PAR-2 and MRGPRX2, as well as the mediator tryptase, following infection. Additionally, levels of the pruritogenic cytokine IL-31 and periostin, a molecule that may promote IL-4, IL-13 and IL-31 expression were elevated, as were substance P and its receptor NK-1R. In addition, the expression of histamine, the primary mediator of the histaminergic itch pathway, was also increased. Notably, reduced expression of β tubulin III across all time points suggests direct mite-induced neuroinflammation. Our findings may serve as a valuable basis for future drug development efforts focused on specific treatment of scabies-associated itch.

**Conclusions/Significance:**

Our data suggest that both the histaminergic and non-histaminergic pathways may be involved in scabies itch. Further research is needed to better understand the complex interplay between these pathways and to identify therapeutic targets for scabies-related itch.

## Author summary

Scabies is a highly prevalent parasitic skin disease globally. Its hallmark symptom is intense pruritus (itching), which can persist for weeks after antiparasitic treatment. Chronic scratching due to this itch compromises the skin barrier, promoting secondary bacterial infections that can lead to severe and potentially life-threatening complications. Despite its clinical burden, the molecular mechanisms underlying scabies-associated itch are not well understood, and no targeted anti-itch therapies currently exist. In this study, we investigated the molecules and pathways associated with scabies itch using a porcine model of scabies, which closely resembles human disease. Our findings suggest that multiple itch pathways are activated during scabies infection. Further research is needed to identify potential drug targets within these pathways and to validate these in human studies for the development of novel scabies itch therapies.

## Introduction

With an annual global prevalence of 100–300 million cases, scabies is among the most common dermatological conditions worldwide [1,2], with an incidence surpassing that of melanoma and keratinocyte carcinoma [3,4]. This highly contagious neglected tropical disease is caused by the parasitic mite *Sarcoptes scabiei* and poses a significant public health burden, particularly in overcrowded and resource-limited settings. Intense pruritus (itching) is the hallmark symptom of scabies, to the extent that the disease is colloquially referred to as “the itch” [5]. It affects more than 90% of patients regardless of age [6], and is more severe than itch associated with atopic dermatitis, non-atopic eczema, or urticaria [7], with a mean score of 7.2/10 on the Visual Analogue Scale (VAS), and a corresponding 6.2/10 VAS score for sleep disturbance [8]. Pruritus typically emerges 4–6 weeks after primary infection and as early as 24 hours following reinfection [9]. Even after successful treatment, itch frequently continues for weeks or develops into a chronic condition. [10,11].

The impact of scabies on quality of life is profound [12], and the itch contributes significantly to social stigma. Moreover, the complications resulting from pruritus remain underappreciated. Scratching disrupts skin integrity, allowing opportunistic bacteria such as *Staphylococcus aureus* and Group A *Streptococcus* to invade. Mite-derived secretory proteins further promote bacterial colonisation [13,14], leading to superinfection of lesions. These secondary infections can escalate to serious complications, including pyoderma [15–19] necrotizing soft tissue infections [20,21], rheumatic fever, and rheumatic heart [22] and renal diseases [22]. Standard scabies treatments, such as permethrin and ivermectin, do not alleviate itch. Furthermore, antihistamines (H1 antagonists) are largely ineffective against scabies-related pruritus [10] and systemic corticosteroids, while often misused, are contraindicated [23]. Currently, there are no approved therapies specifically targeting scabies-induced itch, largely due to an incomplete understanding of its underlying pathobiology. Advancing this understanding is essential for developing effective targeted treatments.

Itch is a common and distressing sensory symptom associated with inflammatory skin diseases, systemic disorders, and neuropathic conditions. Two major neuronal pathways mediate itch: histaminergic and non-histaminergic. Histaminergic nerves express histamine receptors, primarily H1R and H4R, which are activated by histamine, whereas non-histaminergic nerves are triggered by diverse pruritogenic mediators through their respective receptors [24]. Scabies itch has been suggested to be mediated by non-histaminergic itch fibers [10]. Non-histaminergic itch is driven by complex interactions between keratinocytes, immune cells, and specific sensory neurons. Key mediators include Protease-Activated Receptors (PARs) and Mas-Related G Protein–Coupled Receptors (MRGPRs). PAR-2 is widely expressed in skin cells including keratinocytes, dendritic cells, mast cells, macrophages, and neurons [25,26], while MRGPRs are mainly restricted to dorsal root ganglia (DRG) and sensory nerve endings - except MRGPRX2, which is also present on mast cells and keratinocytes [27]. Upon PAR-2 activation, keratinocytes release Thymic Stromal Lymphopoietin (TSLP), which stimulates fibroblasts to secrete substance P. Substance P then promotes differentiation of macrophages into the M2 phenotype and induces IL-31 production - a key non-histaminergic pruritogen [28,29]. Additionally, substance P and proinflammatory cytokines enhance MRGPRX2 expression, further amplifying the itch cascade [27].

Two prior studies have reported increased expression of PAR-2, tryptase, TSLP, periostin, TRPA1, TRPV1, and IL-31 in scabies lesions [10,29]. They also documented increased epidermal nerve fiber density and reduced histamine levels in the skin [10]. Even though increased expression of TRPA1 has been detected in one study, involvement of MRGPRX2 in scabies itch is not explored [10]. Notably, these two studies have only looked at a single time point of the infection, and not over the course of infection. Given that the expression of pruritogenic mediators likely varies with infection progression and severity, a longitudinal analysis is essential. Human studies are logistically and ethically challenging, particularly with early-stage or repeated sampling. As such, porcine skin serves as the most suitable *ex vivo* model, due to its anatomical, immunological, and pharmacokinetic similarities to human skin [30,31]. Therefore, this study utilised a modified version of the established porcine scabies model [32] to investigate the pathophysiology of scabies-associated itch across multiple time points.

## Methods

### Ethics statement

The study was approved by the University of Queensland and QIMR Berghofer Animal Ethics Committees, 2021/AE001015 and P630. Animal handling, care and the procedures involved in this study were followed according to the Animal Care and Protection Act and in compliance with the Australian code of practice for the care and use of animals for scientific purposes, outlined by the Australian National Health and Medical Research Council (NHMRC).

### Porcine scabies model and skin biopsies

The porcine scabies experimental model was established following previously published protocols, with the exception of dexamethasone administration [32]. In this study, dexamethasone was omitted to preserve the pigs’ natural immune responses. Three female sibling piglets (*Sus scrofa domesticus*, Large White breed), two weeks of age and with no prior antiparasitic treatment, were experimentally infected with *Sarcoptes scabiei* var. *suis*. Mite-infested skin crusts were harvested from donor pigs maintained under a long-term porcine scabies model. These crusts were dissected into ~0.5 cm² fragments, and an equal number of pieces were inserted into both ear canals of each piglet. To ensure successful infection and prevent dislodgement, the ears were wrapped with cohesive bandages for 30 minutes. Skin punch biopsies (3 mm) were collected from both ears at weeks 0 (baseline), 2, 4, 8, 12, and 20 post-infection. Biopsies were fixed in 10% neutral-buffered formalin and embedded in paraffin for subsequent histological analysis.

### Immunohistochemistry

Paraffin embedded 4 μm consecutive sections were used to localise individual itch markers using immunohistochemistry. Specific polyclonal antibodies were purchased from Abcam (Cambridge, UK), including anti-PAR2 rabbit antibody (ab138479), anti-MGPRX2 rabbit antibody (ab237047), anti-β tubulin III rabbit antibody (ab18207), anti-periostin rabbit antibody (ab152099), anti-IL-31 rabbit antibody (ab62579), anti-NK-1R rabbit antibody (ab219600), and anti-substance P mouse antibody (ab14184). The anti-tryptase antibody (M7052) was purchased from Dako (Glostrup, Denmark), and the anti-histamine antibody (H7403) was purchased from Sigma-Aldrich (St Louis, USA).

The sections were deparaffinised, rehydrated and rinsed with tris buffered saline (TBS). Antigen retrieval was performed by microwaving slides in antigen retrieval buffer until just boiling (approximately 37 seconds at 100% power), followed by 15 minutes at 10% power. Antigen retrieval buffers, 10mM Tris-EDTA at pH 9.0 (PAR-2 and IL-31), 1X Biocare Medical Diva Decloaker (Pacheco, USA) (β tubulin III, tryptase and histamine) or Dako (Glostrup, Denmark) at pH 6.0 (MRGPRX2, periostin, NK-1R and substance P) were used. Slides were cooled to room temperature and washed three times with TBS containing 0.05% Tween-20 (TBST). Non-specific binding was blocked using Background Sniper (Biocare Medical, Pacheco, USA) for 20 minutes at room temperature and incubated overnight at 4°C with or without (negative controls) the respective primary antibodies in Background Sniper. Primary antibody dilutions were; 1:50 for IL-31 and substance P, 1: 100 for PAR-2, MRGPRX2, periostin and tryptase, 1:200 for histamine, 1:250 for NK-1R and 1:500 for β tubulin III. Subsequent steps were performed at room temperature and washes were performed 3 times with PBST unless otherwise stated. Following primary antibody incubation, the slides were washed and incubated with respective secondary antibodies at 1:400 dilution for 1 hour. Alexa Flour 555 (tryptase, substance P, PAR-2, MRGPRX2, β tubulin III, periostin, IL31 and NK-1R) or Alexa Fluor 647 (histamine) (Life Technologies, Carlsbad, USA) were used as secondary antibodies. Subsequently, slides were washed and stained with DAPI nuclear stain (Sigma-Aldrich, 1:35,000 in PBS) for 10 minutes. After a final wash, coverslips were mounted using Dako Fluorescence Aqueous Mounting Medium (Glostrup, Denmark) and scanned using an Aperio ScanScope FL fluorescence slide scanner (Aperio Technologies, Vista, USA). The images were analysed using QuPath (v 0.5.0) quantitative digital pathology software [33].

### Data acquisition and analysis

Image analysis was performed using QuPath software (version 0.5.0). Regions of interest for expression analysis were defined using the pixel classification module with fluorescent signal thresholding. The total tissue and epidermal areas were first delineated, with further refinement using the brush tool to ensure accurate boundary identification. The epidermal area was then duplicated and subtracted from the whole tissue area, isolating the analysis to the dermal and epidermal layers of the porcine skin. For markers localised exclusively to the dermis, the epidermal area was excluded from the analysis. The positive cell detection module was employed to differentiate positive and negative cells based on thresholds set for DAPI (nuclear staining) and AF555 (or AF647 for histamine). β tubulin III and periostin expression were quantified using the pixel classification module with an AF555 signal threshold, allowing the inclusion of extracellular expression in the analysis. Thresholds for each marker were visually determined and kept consistent across the different infection time points to ensure uniformity and comparability. The established thresholds and analysis parameters for each marker were incorporated into a script to enable batch processing for high-throughput analysis.

The number of positive cells was analysed using Poisson regression with log (area mm^2^) used as offset, estimating and comparing rates of number positive per mm^2^ of tissue area. The models also accounted for overdispersion and used Firth Bias-adjusted estimates, and accounted for differences between pigs and between ears of each pig. These models were used to analyse differences in rates between weeks, starting with an overall week effect test and then comparing contrasts between each week with and week 0, using Dunnett’s multiple comparison adjustment. Percentage of log positive area was analysed using least squares regression.

## Results

### Scabies infection

Despite the omission of immunosuppressing drugs, all three pigs were successfully infected with scabies, and the infection progressed to low-grade crusted scabies by 6 weeks post-infection. Successful infection was confirmed by the presence of mites in H&E stained skin biopsy sections and crusted scabies was confirmed by appearance of skin crusts using clinical photographs.

### Immunohistological localisation of itch mediators

**Fig 1:**
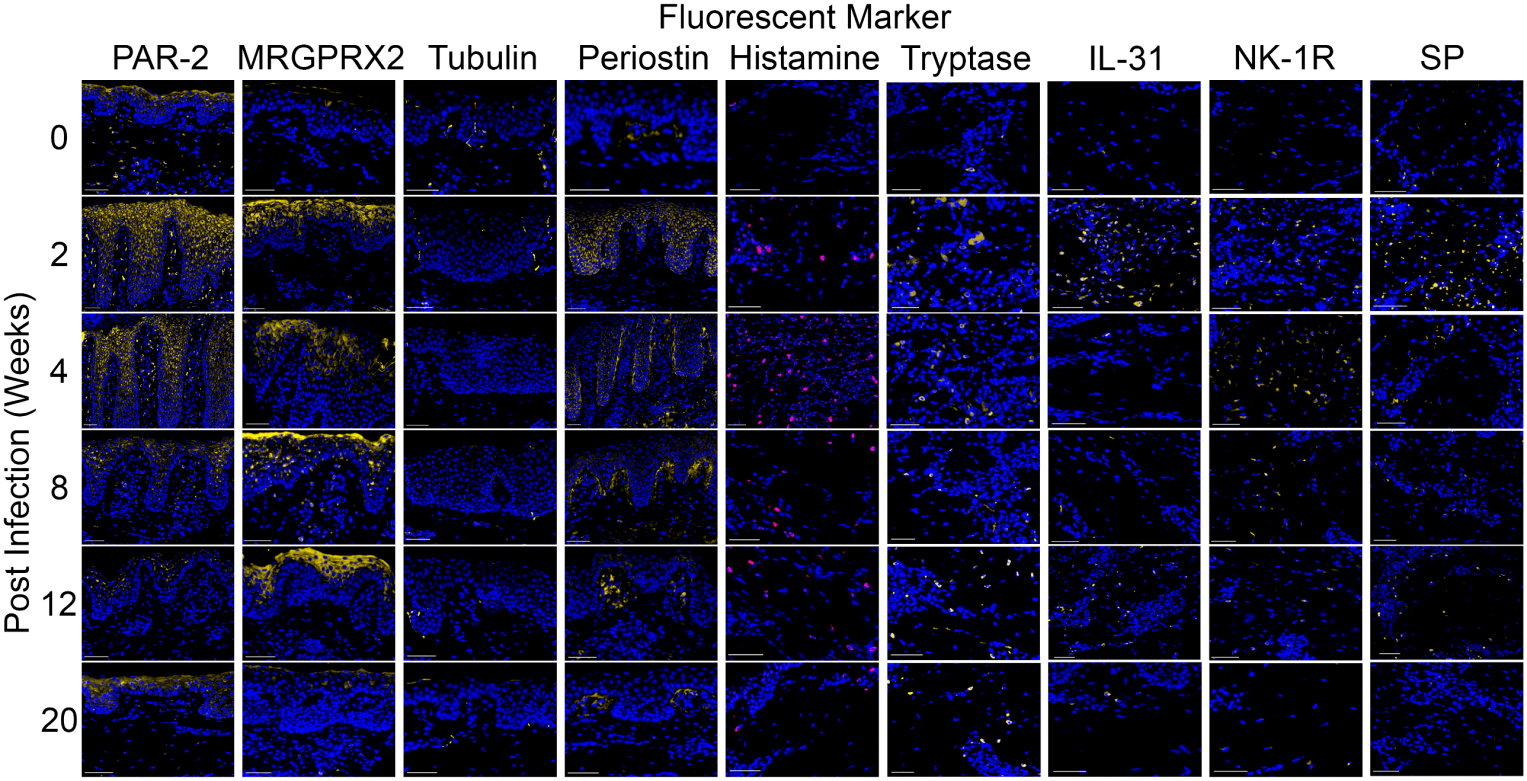
Immunohistological localisation of itch mediators in porcine skin biopsies. Localisation of PAR-2, MRGPRX2, β tubulin III, periostin, histamine, tryptase, IL-31, NK-1R, and substance P are depicted with fluorescence signals in yellow or pink (from left to right). Nuclear staining (DAPI) is shown in blue. Tissue samples from pre-infection (week 0) and weeks 2, 4, 8, 12, and 20 post-infection are shown (top to bottom). Crusted scabies developed by 6 weeks. Images were captured using the Aperio ScanScope FL fluorescence slide scanner (Aperio Technologies, Vista, USA). (Scale bar = 50 µm).

Although all of the antibodies used in this study were raised against human proteins, they all cross-react with porcine versions of the proteins. PAR-2 and MRGPRX2 were localised to keratinocytes and dermal cells (Fig 1, columns 1 and 2). The expression of β tubulin III was observed in the epidermal nerve fibers (Fig 1, column 3). Periostin was localised in keratinocytes and both extracellularly and intracellularly within the dermis (Fig 1, column 4). Histamine and tryptase were primarily localised in mast cells in the dermis, with some mast cells expressing histamine only or tryptase only, while most co-expressed both markers (Fig 1, columns 5 and 6). IL-31, NK-1R, and substance P were exclusively localised in the dermis (Fig 1, columns 7–9).

### Expression profiles of scabies itch mediators

**Fig 2:**
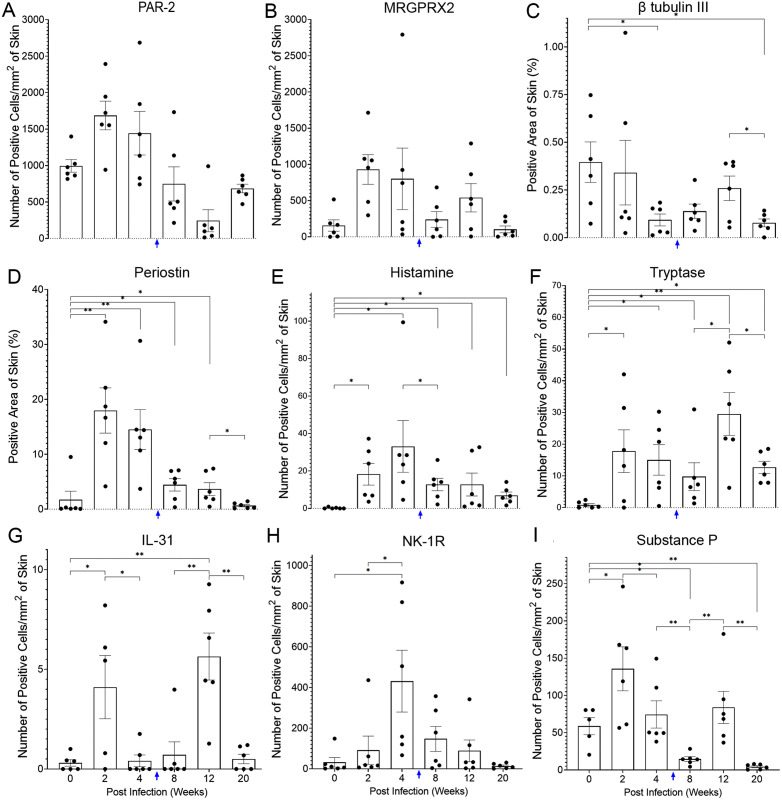
Expression profiles of itch mediators during scabies infection. Expression of PAR-2 **(A)**, MRGPRX2 **(B)**, β tubulin III **(C)**, periostin **(D)**, histamine **(E)**, tryptase **(F)**, IL-31 **(G)**, NK-1R (H) and substance P (I) in pre-infection (week 0) and week 2, 4, 8, 12, and 20 post-infection are shown. Ordinary scabies was developed into crusted scabies at week 6 (blue arrow) and error bars indicate mean ± SEM.

Tissue PAR-2 and MRGPRX2 expression was increased from pre-infection to 2 weeks post-infection followed by a gradual decrease until 20 weeks post-infection (Fig 2A and 2B and S1 and S2 Tables). Differences between weeks were not statistically significant (P = 0.7303 and P = 0.6654 respectively). Compared to pre-infection, β tubulin III expression decreased during infection (Fig 2C and S3 Table). This reduction is statistically significant at week 4 and 20 post-infection (P < 0.05). Periostin expression increased at 2 weeks post-infection (P < 0.0001) and maintained a higher expression at 4 weeks (P < 0.0001), 8 weeks (P = 0.0001) and 12 weeks (P = 0.0003) post-infection when compared to pre-infection. However, its expression at 20 weeks post-infection was similar to pre-infection and its decrease from week 12–20 was statistically significant (P < 0.05) (Fig 2D and S4 Table). Interestingly, expression of histamine and tryptase had the same trend with significantly higher expression at all tested time points during the scabies infection (P < 0.05) and with the highest expression at week 12 post-infection (Fig 2E and 2F and S5 and S6 Tables). IL-31 expression was increased at weeks 2 and 12 post-infection (P < 0.05) (Fig 2G and S7 Table). NK-1R expression was increased until 4 weeks post-infection (P < 0.05, at week 2 and 4) and then gradually decreased until week 20 post-infection (Fig 2H and S8 Table). Substance P expression didn’t show a particular trend during scabies infection as its expression at week 2 and 8 were significantly higher (P < 0.05) and it was significantly lower at week 20 post-infection (P < 0.0001) (Fig 2I and S9 Table).

## Discussion

Itch is a sensory experience triggered by the activation of proprioceptors in peripheral neurons, which transmit signals to the central nervous system, resulting in the compulsion to scratch. Various mediators and ion channels play a crucial role in this process. These mediators are classified into histaminergic and non-histaminergic categories, with some playing a role in both pathways. Notable mediators include histamine, tryptase, cytokines (e.g., IL-4, IL-13, IL-31, IL-33, TSLP), neurotransmitters (e.g., substance P, CGRP, vasoactive intestinal peptide, neuropeptide Y, NBNP, endothelin 1, and gastrin-releasing peptide), and neurotrophins (e.g., nerve growth factor, brain-derived neurotrophic factor). In addition, ion channels include; voltage-gated sodium channels, transient receptor potential vanilloid 1 (TRPV1), transient receptor potential ankyrin (TRPA1), and transient receptor potential cation channel subfamily M (melastatin) member 8 (TRPM8). Itch can be induced by allergens, parasites, and immunological disorders, with scabies being a parasitic skin disease characterised by intense itch.

Scabies itch, however, remains understudied, and there is no molecular data to fully explain the mechanisms behind it. Clinical observations suggest a lack of response to antihistamines, and immunohistological data from two studies indicate that the histamine-independent (non-histaminergic) pathway may be involved in scabies-related itch [10,29]. Due to the challenges of obtaining human biopsy samples, we employed a porcine scabies model. While immunosuppression is typically required for severe infection, we modified the model by omitting immunosuppression, allowing us to observe scabies lesions from week 2 post-infection and crust development by week 6. At this stage of scabies infection pigs generally exhibits high level of scratching behaviour [34,35] and displays gene expression profiles associated with psoriasis and atopic dermatitis with pronounced Th2/17 responses [36].

The key markers in the non-histaminergic itch pathway, PAR-2 and MRGPRX2 receptors, were assessed in porcine scabies. Expression of both receptors was upregulated at weeks 2 and 4 post-infection but showed a gradual decrease thereafter, though these changes were not statistically significant. In contrast, Sanders K.M. *et. al.,* have shown a significantly increased PAR-2 expression in porcine scabies at nine weeks post-infection, however immune suppression with dexamethasone was implicated in that study and it was restricted to a single timepoint observation at 9-week post-infection [10]. PAR-2 and MRGPRX2 receptors are activated by exogenous or endogenous proteins, such as scabies mite excretory proteins (e.g., cysteine proteases, serine proteases, and aspartic proteases) and proteins secreted by immune cells (e.g., tryptase from mast cells). We hypothesise that these proteins are responsible for activating PAR-2 and MRGPRX2, triggering the non-histaminergic itch pathway. Further longitudinal human studies across the infection, treatment, and post-treatment phases are needed to clarify the involvement of these receptors in scabies.

Tryptase, which selectively activates PAR-2, is a known mediator in the non-histaminergic itch pathway. Histamine, on the other hand, is the primary mediator in the histaminergic pathway, activating sensory nerve endings by binding to H1 and H4 receptors. In the skin, histamine is predominantly expressed in mast cells, while tryptase is considered mast cell-specific [37]. Therefore, in the absence of other mast cell-specific markers, tryptase can be used as a reliable indicator of mast cells. In our study, the expression of both mediators was significantly increased at all timepoints compared to pre-infection, suggesting that scabies itch may involve both the histaminergic and the non-histaminergic pathways. This observation aligns with the findings of Sanders *et. al*., who noted increased tryptase expression in scabies lesions, although no change in histamine expression was reported in their study. Notably, histamine expression was reduced in human scabies lesions compared with non-lesional skin [10]. These findings suggest that future in-depth studies are needed to explore the interplay between these two itch pathways in scabies.

Substance P (SP) is a neurotransmitter secreted by primary sensory nerves into the skin upon neuronal activation either through histaminergic or non-histaminergic itch pathway, and SP is a modulator in the mammalian nervous system. SP is an endogenous ligand for the neurokinin 1 receptor (NK-1R) on nerve endings, keratinocytes, immune cells and fibroblasts in the skin activating signal transduction pathways within the cell. SP binds to NK-1R and also to MRGPRX2 receptors [38], thereby playing a role in itch. In addition, SP mediates IgE-independent mast cell degranulation, thereby inducing inflammation [38]. Increased SP expression has been observed in chronic, non-infectious pruritic conditions such as psoriasis and atopic dermatitis [39]. Our study is the first study to examine SP and NK-1R expression in scabies. We observed that SP expression was significantly elevated two weeks after scabies infection, indicating that SP contributes to the transmission of the itch signal through NK-1R and MRGPRX2 activation, resulting in continuous itch induction via mast cell degranulation. We also observed a significant increase in NK-1R expression at week 4, followed by a gradual decrease. NK-1R plays a predominant role in transmission of itch signal by peripheral sensory nerves where SP released by pre-sympathetic terminals of peripheral nerves binds to NK-1R in the post-sympathetic terminals of neurones of the spinal cord [38]. Similar to SP, NK-1R expression is also increased in psoriasis and atopic dermatitis on the lesional site compared to non-lesional site [39].

Among cytokines that play a role in itch, IL-31, often referred to as the “itchy cytokine,” is secreted by Th2 cells and dendritic cells. IL-31 is a member of IL-6 family of cytokines that is secreted by Th2 type cells and dendritic cells and activates IL31RA/OSMRβ expressing dorsal root ganglia neurones and keratinocytes. IL-31 association with IL31RA on sensory nerve endings results in sending itch signals to central nervous system [40]. Over expression of IL-31 has been detected in other itchy dermatoses like atopic dermatitis and prurigo nodularis [41,42]. In our study, IL-31 expression was significantly increased at weeks 2 and 12 post-infection (P < 0.05), which supports the idea that IL-31 plays a role in scabies-related itch. A study that looked at four scabies patients has also shown an increased infiltration of IL-31 + cells in the scabies lesions compared to non-lesional site and more than half of those cells were CD68+ M2 macrophages [29]. Furthermore, it is suggested that the periostin and TSLP expressed in scabies potentially involved in IL-31 generation from M2 macrophages [29]. Our findings align with this hypothesis, as periostin expression was significantly elevated from weeks 2–12. Further studies are needed to explore the protease-TSLP-periostin axis in IL-31 production by macrophages.

Neurotrophins are important mediators in itch and they are non-pruritogenic growth factors that have shown to be increased in pruritogenic diseases like atopic dermatitis [43]. While their expression in scabies is not clear, these growth factors could potentially enhance sensory nerve growth, which could have a role in chronic itch. β tubulin III is a protein that plays a crucial role in the growth and maintenance of neuronal structures and is exclusively localised to neurons. Therefore, we measured epidermal nerve fibre density (ENFD) by localising β tubulin III. ENFD has been shown to be increased in human scabies patients [10,29] while no change has been previously observed in porcine scabies [10]. In contrast, ENFD was reduced at all timepoints post-infection in our pigs (albeit statistically significant reduction was seen only in week 4 and in week 20, P < 0.05). This could be due to intense scratching as described in other pruritic dermatoses [44,45] indicative of direct nerve damage by mite and/or scratching. The exclusion of steroids in this study’s porcine model allows for a clearer assessment of scabies mite-induced itch pathway activation. Nevertheless, the lack of scratching data (including onset, severity, and duration), and the absence of clinical and laboratory measures (such as inflammatory markers, antibody profiles, and indicators of hypersensitivity), constitute major limitations. Recognising these constraints, future studies will implement continuous video monitoring to capture detailed behavioural data from individual pigs, and will systematically collect clinical and laboratory parameters. Nevertheless, the established methodology and initial findings of this study provide a foundation for future research into precision treatments for scabies-related itch that target its underlying biological pathways.

## Conclusion

This study provides novel insights into the expression patterns of key itch mediators in scabies using a porcine model. Our findings suggest that both histaminergic and non-histaminergic pathways may be involved in scabies itch. However, the absence of pruritus scores for individual animals in this study limits our ability to correlate these expression patterns with clinical observations. Further research is needed to identify potential therapeutic targets for scabies-related itch and to better understand the complex interplay between these pathways.

## Supporting information

S1 Table Expression of PAR2 (raw data). (XLSX)

S2 Table Expression of MRGX2 (raw data). (XLSX)

S3 Table Expression of β tubulin III (raw data). (XLSX)

S4 Table Expression of Periostin (raw data). (XLSX)

S5 Table Expression of Histamine (raw data). (XLSX)

S6 Table Expression of Tryptase (raw data). (XLSX)

S7 Table Expression of IL-31 (raw data). (XLSX)

S8 Table Expression of NK-1R (raw data). (XLSX)

S9 Table Expression of Substance P (raw data). (XLSX)
